# Dissecting Intra-Tumoral Changes Following Immune Checkpoint Blockades in Intrahepatic Cholangiocarcinoma *via* Single-Cell Analysis

**DOI:** 10.3389/fimmu.2022.871769

**Published:** 2022-04-26

**Authors:** Bao-Ye Sun, Cheng Zhou, Ruo-Yu Guan, Gao Liu, Zhang-Fu Yang, Zhu-Tao Wang, Wei Gan, Jian Zhou, Jia Fan, Yong Yi, Shuang-Jian Qiu

**Affiliations:** Department of Liver Surgery and Transplantation, Liver Cancer Institute, Zhongshan Hospital, and Key Laboratory of Carcinogenesis and Cancer Invasion (Ministry of Education), Fudan University, Shanghai, China

**Keywords:** tumor ecosystem, immunotherapy, ICC, single-cell analysis, data mining

## Abstract

**Purpose:**

To dissect the tumor ecosystem following immune checkpoint blockades (ICBs) in intrahepatic cholangiocarcinoma (ICC) at a single-cell level.

**Methods:**

Single-cell RNA sequencing (scRNA-seq) data of 10 ICC patients for the ICB clinical trial were extracted from GSE125449 and systematically reanalyzed. Bulk RNA-seq data of 255 ICC patients were analyzed. Infiltration levels of SPP1^+^CD68^+^ tumor-associated macrophages (TAMs) were examined by dual immunofluorescence (IF) staining in 264 resected ICC samples. The correlation between SPP1^+^ TAMs and clinicopathological features as well as their prognostic significance was evaluated.

**Results:**

Among the 10 patients, five received biopsy at baseline, and others were biopsied at different timings following ICBs. Single-cell transcriptomes for 5,931 cells were obtained. A tighter cellular communication network was observed in ICB-treated ICC. We found a newly emerging VEGF signaling mediated by PGF-VEGFR1 between cancer-associated fibroblasts (CAFs) and endothelial cells in ICC following ICBs. SPP1 expression was dramatically upregulated, and SPP1^+^ TAM gene signatures were enriched in TAMs receiving ICB therapy. We also identified SPP1^+^ TAMs as an independent adverse prognostic indicator for survival in ICC.

**Conclusion:**

Our analyses provide an overview of the altered tumor ecosystem in ICC treated with ICBs and highlight the potential role of targeting CAFs and SPP1^+^TAMs in developing a more rational checkpoint blockade-based therapy for ICC.

## Introduction

Intrahepatic cholangiocarcinoma (ICC) ranks the second most common primary liver cancer (1). As a highly aggressive and chemotherapy-resistant malignancy, ICC accounts for 10%–15% of primary liver cancer and typically features an extremely low life expectancy of around 1 year (2). Most patients are diagnosed at an advanced stage, missing the opportunity for curative resection (1). Even for those receiving surgical resection, the 5-year survival rate remains dismal (3). Thus, identifying novel therapeutic approaches that significantly prolong the survival of patients with ICC is urgently needed.

Tumors employ multiple tactics to evade immune attack. One dominant mechanism is the upregulated programmed cell death (PD) pathway in the tumor microenvironment, resulting in a defective antitumor immune response. Immunotherapies have revolutionized the anticancer treatment landscape over the past decades (4). Immune checkpoint blockade (ICB) therapy, using monoclonal antibodies targeting immune-inhibitory receptors like PD-1/PD-L1 and CTLA-4, seeks to reactivate the impaired T-cell response against tumor. ICBs have demonstrated promising antitumor activity in several refractory malignancies, including advanced hepatocellular carcinoma (HCC) (5–7). Despite the scarce reports of anti-PD therapy efficacy in ICC, a previous study reported that PD-1 inhibitor pembrolizumab induced robust and durable efficacy in an advanced cholangiocarcinoma case (8). However, clinical trials of anti-PD therapy in advanced bile tract cancer (BTC) have so far failed to show a higher treatment response or clinical benefit compared with standard chemotherapies (9). In the KEYNOTE-158 study, pembrolizumab monotherapy achieved a durable objective response in 6% to 13% of patients with advanced BTC, whereas most patients did not obtain more survival benefits (10). These findings suggest that a limited proportion of patients with ICC can benefit from immunotherapy due to innate or adaptive tumor resistance to ICBs, highlighting the necessity for combining other treatment strategies such as multityrosine kinase inhibitor (TKI), chemotherapy (11, 12), and other novel targets.

Therefore, it is crucial to elucidate the mechanism underlying the poor response of ICC to anti-PD therapy. The tumor immune microenvironment could affect the treatment efficacy of ICBs (13). Dissecting the intra-tumoral changes following ICBs in ICC could help find more effective therapeutic targets and enhance the antitumor efficacy of immunotherapy. Single-cell RNA sequencing (scRNA-seq) has emerged as a powerful tool for investigating the complex cellular components in the liver tumor microenvironment (14–18), and these single-cell datasets require further mining.

Herein, we performed a comprehensive comparison of the ICC ecosystem before and during ICB treatment *via* reanalyzing a publicly available scRNA-seq dataset (17). As expected, ICBs remarkably affected the tumor microenvironment landscape in ICC. We observed an increased number as well as enhanced strength of cellular interactions in ICB-treated ICC, especially between malignant cells and cancer-associated fibroblasts (CAFs). CAFs could promote angiogenesis through directly interacting with tumor-associated endothelial cells (TECs) *via* VEGF signaling in ICC following ICB therapy. Moreover, SPP1 expression was dramatically upregulated in tumor-associated macrophages (TAMs) following ICBs, and SPP1^+^ TAMs correlated with adverse clinical outcomes in an independent cohort of 264 patients with ICC. Our analyses provided insights into the altered tumor ecosystem of ICC treated with ICBs, which might aid in the development of rational strategies to surmount the tumor resistance to checkpoint blockade immunotherapy.

## Materials and Methods

### Study Design and Patient Selection

This study included three ICC cohorts. (1) The first cohort included 10 ICC patients (ICB cohort). We extracted the single-cell data of 10 patients with ICC from Gene Expression Omnibus (GEO) dataset GSE125449 (17) for subsequent analysis and divided them into two groups based on whether they were treated with ICBs. (2) The second cohort enrolled 255 patients from the FU-iCCA cohort. The bulk RNA-seq data of 255 patients with ICC from Zhongshan Hospital, Fudan University (FU-iCCA cohort) (19), were analyzed and are available in the biosino NODE database (NODE database: OEP001105). (3) The third cohort recruited a cohort of 264 consecutive patients undergoing curative resection for ICC from 2012 to 2017 in Zhongshan Hospital (ZSH cohort). All enrolled patients received no prior anticancer therapy and met the inclusion criteria detailed before (20). All tissue specimens of the ZSH cohort were formalin-fixed and paraffin-embedded. The baseline clinicopathological features of the ZSH cohort are detailed in **Table 1**. Serological tests, including CA199, carcinoembryonic antigen (CEA), and γ-glutamyl transpeptidase (GGT) levels, were performed within 3 days before surgery. The clinical stage was evaluated according to the American Joint Committee on Cancer (AJCC) 8th edition (21).

**Table 1:** Correlation between SPP1^+^ TAMs and clinical features of patients enrolled in the ZSH cohort.

Characteristics	Patients		SPP1^+^ TAMs		
	No.	%	Low	High	*P*
All patients	264	100	149	115	
Sex					0.741
Female	104	39.4	60	44	
Male	160	60.6	89	71	
Age					0.700
≤60	125	47.3	69	56	
>60	139	52.7	80	59	
Liver cirrhosis					0.183
No	191	72.3	103	88	
Yes	73	27.7	46	27	
Microvascular invasion					0.081
No	189	71.6	113	76	
Yes	75	28.4	36	39	
LN metastasis					**0.048**
No	212	80.3	126	86	
Yes	52	19.7	23	29	
Tumor number					**0.001**
Single	201	76.1	125	76	
Multiple	63	23.9	24	39	
Tumor size					**0.004**
≤5 cm	118	44.7	78	40	
>5 cm	146	55.3	71	75	
Tumor differentiation					0.699
I–II	93	35.2	51	42	
II–III	171	64.8	98	73	
CA199					**0.009**
≤37 U/ml	118	44.7	77	41	
>37 U/ml	146	55.3	72	74	
CEA					**0.002**
≤5 ng/ml	193	73.1	120	73	
>5 ng/ml	71	26.9	29	42	
GGT					**0.005**
≤60 U/l	143	54.2	92	51	
>60 U/l	121	45.8	57	64	
AJCC 8th					0.089
I–II	208	78.8	123	85	
IIIa–IIIb	56	21.2	26	30	

LN, lymph node; CEA, carcinoembryonic antigen; GGT, γ-glutamyl transpeptidase; AJCC, American Joint Committee on Cancer.

Bold indicated statistical significance.

### Single-Cell Data Analysis

The Seurat v4 (version 4.0.4) R package was used to analyze the scRNA-seq data (22). After normalization and principal component analysis (PCA) on the highly variable genes (k = 2400), we selected the top 20 PCs with a resolution parameter equal to 0.5 for the clustering of all cells and the top 12 PCs with a resolution parameter of 0.6 for the clustering of T cells. The annotation of major cell types was performed according to the original article except that the unclassified cluster cells were removed. The annotation of T-cell subtypes was performed according to their highly expressed marker genes or well-known functional genes. Copy number variation (CNV) analysis was conducted among the annotated cell types *via* the inferCNV R package (23) to identify malignant cells with endothelial cells set as control. The function AddModuleScore in Seurat was used to calculate the scores of SPP1^+^ TAM gene signatures and C1QC^+^ TAMs gene signatures defined in Zhang et al.’s study (24). Scores of “classically activated” (M1) macrophage and “alternatively activated” (M2) macrophage gene signatures (25) were also calculated, respectively. These gene signatures are listed in **Table S1**.

### Cell Developmental Trajectory

The cell lineage trajectory of CD8^+^ and CD4^+^ T lymphocytes was inferred and visualized separately using Monocle2 (26).

### Cell–Cell Interaction Analysis

The CellChat R package (27)was utilized to infer, visualize, and analyze intercellular communication among different cell types based on scRNA-seq data. Differential ligand–receptor interactions and specific signaling pathways were also identified using CellChat.

### Differentially Expressed Gene Analysis

Differential gene expression analysis of malignant cells as well as TAMs before and after ICB treatment was conducted using the “FindMarkers” function in the Seurat package, with a log-scaled fold change ≥0.25 and P value < 0.05. The EnhancedVolcano R package was used to visualize the differentially expressed genes. Differentially expressed genes of TAMs passing the criteria are shown in **Table S2**.

### Bulk RNA-Seq Data and Immune Cell Infiltration Estimation

In the FU-iCCA cohort, the bulk RNA-seq gene expression data were log2(TPM+1) transformed, and the mean expression of 10 highest upregulated genes (SPP1, S100A9, NUPR1, S100A8, RETN, MARCO, FCGR3A, MT2A, TMEM176B, APOE) in TAMs following ICBs was defined as the SPP1^+^ TAM gene signature score in the FU-iCCA cohort. We used CIBERSORT (28) to estimate the immune cell infiltration level based on bulk RNA-seq data.

### Gene Set Enrichment Analysis

Gene Set Enrichment Analysis (GSEA) (29) was performed to investigate the difference in hallmark gene sets of malignant cells and TAMs before and after ICB treatment.

### Tissue Microarray and Immunofluorescence Staining

For the ZSH cohort, tissue microarrays (TMAs) of 264 ICC specimens were constructed as previously described (20, 30).The immunofluorescence (IF) staining of TMAs was performed according to the procedures detailed before (20). The slides were incubated with a CD68 primary antibody (dilution 1:400, #76437, CST, Danvers, USA), followed by an anti-rabbit Alexa Fluor 594-conjugated secondary antibody. Subsequently, the slides were incubated in SPP1 polyclonal Goat IgG (dilution 1:100, #AF1433, R&D, Minneapolis, USA), and anti-goat Alexa Fluor 488-conjugated secondary antibody (Thermo Fisher Scientific, Waltham, MA, USA) sequentially.

### Quantification of SPP1^+^ TAMs

The panoramas of IF staining of all slides were scanned and then evaluated. For each patient, three independent microscopic fields (×400) of a macrophage-enriched tumoral area were selected and counted manually by 2 investigators blinded to patient information. Discrepancies between investigators were resolved together. The cutoff values of SPP1^+^ TAMs were determined by R for optimal survival separation.

### Survival Analysis

For the FU-iCCA cohort, the given 10-gene signature score was calculated in 239 samples with complete follow-up data. For the ZSH cohort of 264 ICC patients, postoperative surveillance was carried out at regular intervals of 2 to 3 months. Overall survival (OS) was defined as the interval from surgical resection to death. Recurrence-free survival (RFS) was the span from surgery to recurrence. The last follow-up of all enrolled patients was censored on December 31, 2020. We analyzed the association of SPP1^+^ TAMs with OS, as well as RFS in the ZSH cohort. The samples were divided into high and low groups based on the optimal cut point determined by R function surv_cutpoint. Kaplan–Meier survival curves were plotted by the Survminer package.

### Statistical Analysis

Comparisons of gene signature expression between two groups of cells were performed using unpaired two-tailed Wilcoxon rank-sum tests. Associations between SPP1^+^ TAMs and baseline clinicopathological variables in the ZSH cohort were evaluated using the chi-squared test. All statistical analyses were performed using R version 4.1.2. Univariate and multivariate analyses were conducted using the Cox proportional hazard model implemented in the R package survival. All figures were plotted using R, and statistical significance was defined as p < 0.05.

## Results

### Single-Cell Atlas of the Tumor Ecosystem in ICC

**Figure 1:**
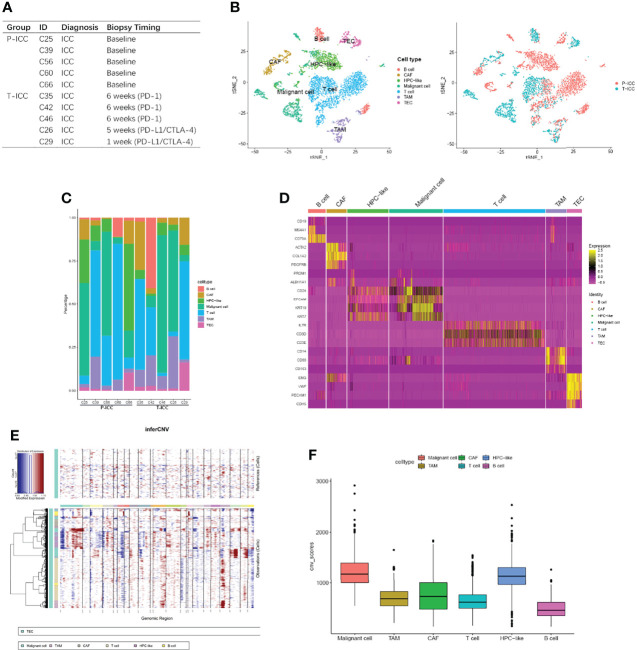
Comprehensive cellular overview of the human ICC ecosystem. **(A)** Table showing information of 10 ICC patients, with five receiving needle biopsy or resection (C60) at baseline and the other five biopsied at different timings during ICB therapy. **(B)** t-SNE plot showing identification of 5,931 single cells colored by cell types (left) and cell origins from P-ICC or T-ICC by color (right). **(C)** Histogram indicating the fraction of cell types in each sample. **(D)** Heatmap showing the top DEGs (Wilcoxon test) in each cell type. **(E)** Heatmap representing the CNV analysis, inferred from the single-cell data. **(F)** Box plots illustrating the CNV scores for each cell type. CNV, copy number variation; DEGs, differentially expressed genes; ICC, intrahepatic cholangiocarcinoma; tSNE, t-distributed stochastic neighbor embedding.

ScRNA-seq data of 10 ICC patients for ICBs clinical trial were extracted from GSE125449 and reanalyzed. Among these patients, five received needle biopsy or resection(C60) at baseline, while the other five patients were biopsied at different timings during the ICB treatment (**Figure 1A**). Core needle or resected tumor biopsies at baseline from primary ICC are referred to as P-ICC, whereas tumor biopsies from ICC samples treated with ICBs (PD-1 or PD-L1/CTLA-4) are denoted as T-ICC. Single-cell transcriptomes for 5,931 cells were obtained. We identified and visualized seven clusters using the T-distributed stochastic neighbor embedding (t-SNE) method. Consistent with the original report, seven distinct cell types were defined using known marker genes: B cells (CD19, MS4A1, CD79A), CAFs (ACTA2, COL1A2, PDGFRB), cells expressing hepatic progenitor cell markers (HPC-like; PROM1, ALDH1A1, CD24), malignant cells (EPCAM, KRT19, KRT7), T cells (IL7R, CD3D, CD3E), TAMs (CD14, CD68, CD163), and TECs (ENG, VWF, PECAM1, CDH5) (**Figures 1B, D** **)**. These cell types were shared at varying ratios among these patients, revealing inter-tumoral heterogeneity of cellular compositions in ICC (**Figure 1C**). Thereafter, inferred CNV analysis revealed that CNV scores of malignant cells were higher than those of other cell types (**Figures 1E, F** **)**.

### Comparison of the Cellular Interaction Between P-ICC and T-ICC

**Figure 2:**
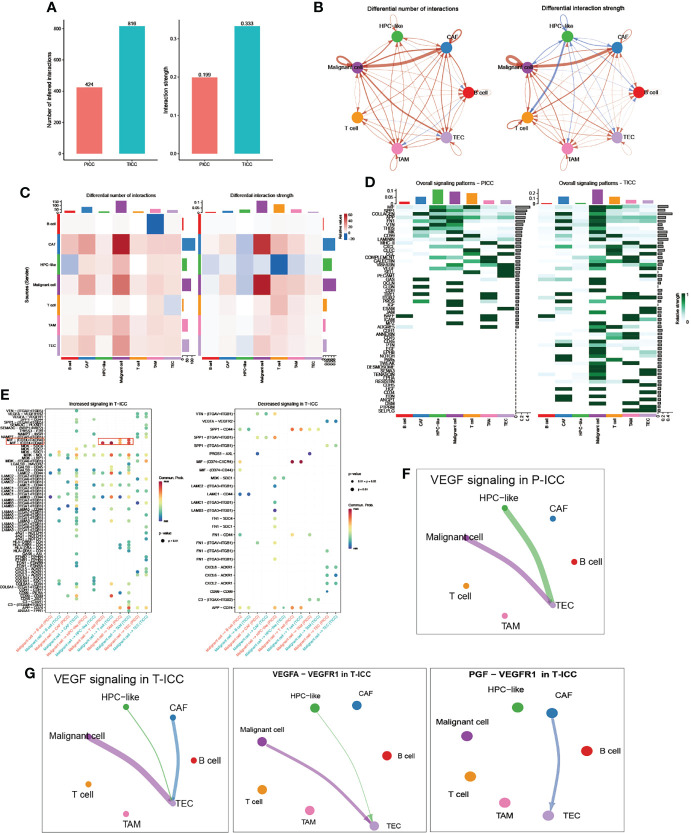
Comparison of cellular interactions between P-ICC and T-ICC. **(A)** Bar plots displaying the sum of number (left) and weights (right) of ligand–receptor interactions between P-ICC and T-ICC. Circle plots **(B)** and heatmap **(C)** showing the differential number (left) and strength (right) of ligand–receptor interactions between distinct cellular components. Clusters are distinguished by colors. Red connecting lines indicated upregulated number or strength. The blue lines indicated reduced number or strength. **(D)** Heatmap showing the differential overall signaling patterns of cell types in P-ICC and T-ICC. **(E)** Dot plots showing the increased (left) and decreased (right) signaling effects of malignant cells on other cell types, respectively. Circle plots showing the VEGF-related signaling networks in P-ICC **(F)** and T-ICC **(G)**. P-ICC, primary ICC; T-ICC, ICB-treated ICC.

Then we investigated the differential communication network mediated by ligand–receptor interactions across all cellular components. Overall cell type interaction analysis exhibited a more intimate interplay in ICB-treated ICC, as evidenced by the increased number as well as enhanced interaction strength in T-ICC compared with those in P-ICC (**Figure 2A**). Specifically, both interaction numbers and interaction strength were remarkably increased between malignant cells and CAFs in T-ICC (**Figures 2B, C** **)**. A comparison of signaling patterns among distinct cell types in P-ICC and T-ICC revealed common signaling pathways like MIF, SPP1, and VTN. Moreover, there were several unique signaling pathways in the T-ICC ecosystem, including CDH1, EGF, NOTCH, PARs, TWEAK, DESMOSOME, SEMA3, CDH5, HGF, CD34, and EDN (**Figure 2D**).

Differential analysis in all ligand–receptor pairs of malignant cells and other cell types demonstrated a distinct pattern between P-ICC and T-ICC (**Figure 2E**). Notably, we found an increased signaling of MIF-(CD74+CD44) in T-ICC between malignant cells and TAMs or T cells, which has been well documented to promote cancer progression (31–34). Moreover, blocking MIF-CD74 signaling could restore the antitumor immune response against metastatic melanoma (35) and MIF inhibitors represented as a potential strategy to overcome resistance to ICB therapy in melanoma (36).

Since VEGF inhibitors, especially when combined with immunotherapy, demonstrated well the clinical efficacy in several caners including HCC (37–39), we compared the VEGF signaling network between P-ICC and T-ICC ecosystems (**Figures 2F, G** **)**. Strikingly, we found a newly emerging VEGF signaling between CAFs and TECs, which was not observed in P-ICC. Further ligand–receptor pair analysis of VEGF signaling indicated that the PGF-VEGFR1 pair mediated the crosstalk between CAFs and TECs in T-ICC, while the canonical VEGFA-VEGFR1 ligand–receptor pair mainly accounted for the interaction between malignant cells and TECs (**Figure 2G**). These results suggested that CAFs could promote angiogenesis *via* PGF-VEGFR1 in ICC following ICB therapy, supporting the rationale for targeting VEGF and CAFs in ICC management.

### Landscape of Tumor-Infiltrating Lymphocytes in P-ICC and T-ICC

**Figure 3:**
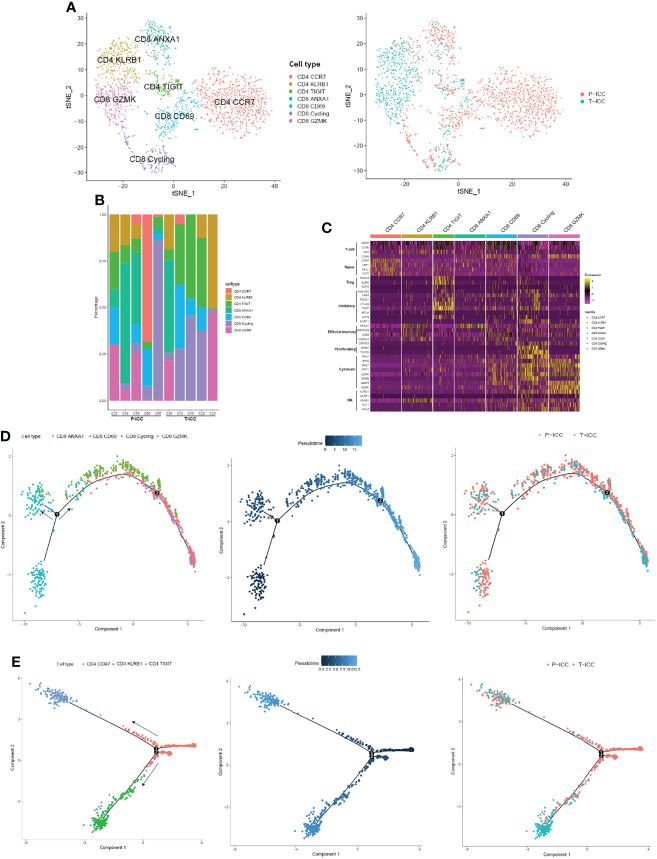
Landscape of infiltrating T cells in P-ICC and T-ICC. **(A)** t-SNE projections of subclustered T cells, labeled in different colors, and T cell origins from P-ICC or T-ICC. **(B)** Bar plots showing the proportion of T-cell subtypes in each sample. **(C)** Heatmap indicating the expression of selected gene sets in T subtypes, including naïve, inhibitory, effector/memory, proliferating, cytotoxic, and cell type. Pseudotime analysis of CD8^+^ **(D)** and CD4^+^ T cells **(E)** from P-ICC and T-ICC. T-cell subtypes and origins are labeled by colors.

An unsupervised reclustering of 2,234 T cells revealed 7 subpopulations, including three subtypes of CD4^+^ T cells (CD4 CCR7, CD4 KLRB1, and CD4 TIGIT) and four clusters of CD8^+^ T cells (CD8 GZMK, CD8 CD69, CD8 ANXA1, and CD8 Cycling) (**Figure 3A**). The proportion of each T-cell subset varied by sample (**Figure 3B**). As shown in **Figure 3C**, CD4 CCR7 cells highly expressed naïve markers CCR7, TCF7, SELL, and LEF1, suggesting these are naïve CD4^+^ T cells. Because CD4 KLRB1 cells showed the highest expression of KLRB1, CD40LG, CD69, and ANXA1, we denoted them as tissue-resident, memory-like T cells. Tregs (CD4 TIGIT) showed an elevated expression of FOXP3 and IL2RA, as well as exhaustion markers (TIGIT, CTLA4, PDCD1, and LAYN).

CD8 CD69 cells represent tissue-resident cells, moderately expressed exhaustion-related markers (TIGIT, LAG3, and PDCD1), and cytotoxic signals (GZMA, NKG7). CD8 GZMK cells displayed a high expression of cytotoxic genes (GZMK, GZMH, GZMA, NKG7, IFNG, and KLRD1) and a low expression of checkpoint genes, suggesting that these cells are cytotoxic T cells. CD8 ANXA1 showed a high expression of ANXA1 and this cluster was not well defined using well-known effector or inhibitory markers. A cluster of CD8^+^ T cells was designated as cycling cells due to overexpressing cell proliferation markers MKI67 and TOP2A. These cycling T cells also showed a high expression of both effector markers GNLY, NKG7, and GZMA and exhaustion markers HAVCR2, LAG3, and TIGIT (**Figure 3C**).

### Trajectory Analysis Revealed Different Distributions of CD4^+^ T Cells in T-ICC

To explore the dynamic CD8^+^ or CD4^+^ T-cell transitions in ICC, we utilized the Monocle 2 to visualize their developmental trajectories. Pseudotime analysis indicated that CD8 cycling cells were at the beginning of the trajectory path with one branch maintaining proliferative capacity, whereas CD8 ANXA1 cells and CD8 GZMK cells were residing at the terminal state along another branch. Then we analyzed the trajectories of CD8^+^ T cells in P-ICC and T-ICC samples separately, and we found that CD8^+^ T cells share the transition trajectory and showed a similar distribution in primary and ICB-treated ICC (**Figure 3D**).

For CD4^+^ T cells, the trajectory path started from CD4 CCR7 cells, with CD4 KLRB1 cells and Tregs (CD4 TIGIT) locating at different terminal ends (**Figure 3E**). CD4^+^ T cells from T-ICC were predominantly distributed at the terminal ends of the transition trajectory pathway, especially CD4 KLRB1 cells, mainly from T-ICC rather than P-ICC. This suggested that this CD4^+^ T subtype was closely related with immunotherapy and its role should be further explored.

### Differential Gene Expression Analysis Between P-ICC and T-ICC Malignant Cells

A differentially expressed gene (DEG) analysis of malignant cells (**Figures S1A, B**) revealed an enrichment of genes involved in cell-cycle-related pathways (e.g., E2F_targets, MYC_targets_V1, and G2M_checkpoint pathways) in T-ICC, whereas the genes upregulated in P-ICC mainly belonged to metabolism-related pathways (e.g., oxidative phosphorylation, fatty acid metabolism, xenobiotic metabolism).

### SPP1^+^ TAM Gene Signatures Were Enriched in T-ICC

**Figure 4:**
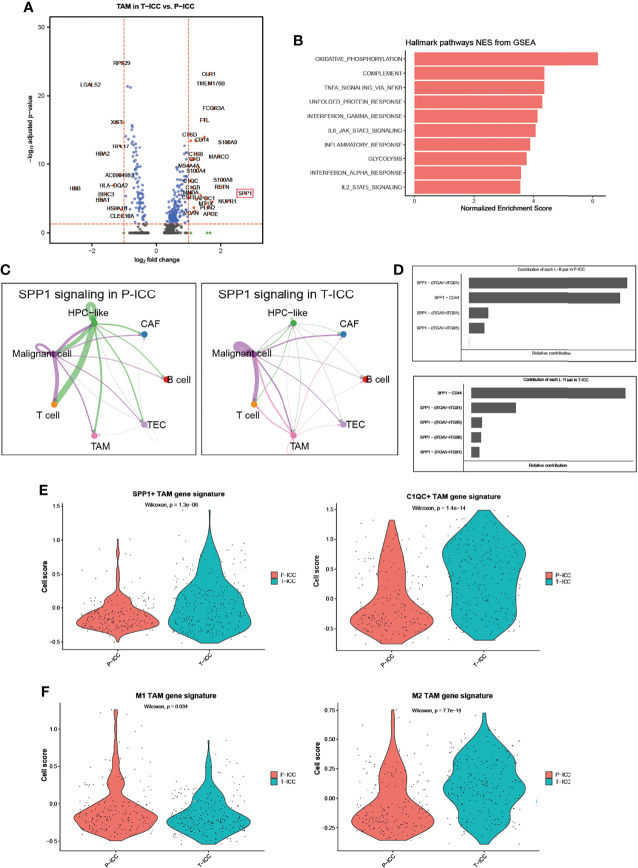
SPP1^+^ TAMs gene signatures were enriched in T-ICC. **(A)** Volcano plot showing differentially expressed genes of TAMs between P-ICC and T-ICC. The most significant genes are indicated in the plots. **(B)** Bar charts showing the enrichment of the hallmark gene set of upregulated genes in TAMs from T-ICC. **(C)** Circle plots showing the SPP1 signaling networks in P-ICC (left) and T-ICC (right). **(D)** Bar plots showing the relative contribution of each ligand–receptor pair in SPP1-related signaling pathways between P-ICC and T-ICC, respectively. Violin plots showing comparison of SPP1^+^ TAM gene signatures and C1QC^+^ TAM gene signature levels **(E)** and M1/M2 gene signatures **(F)** between P-ICC and T-ICC samples. TAMs, tumor-associated macrophages.

**Figure 5:**
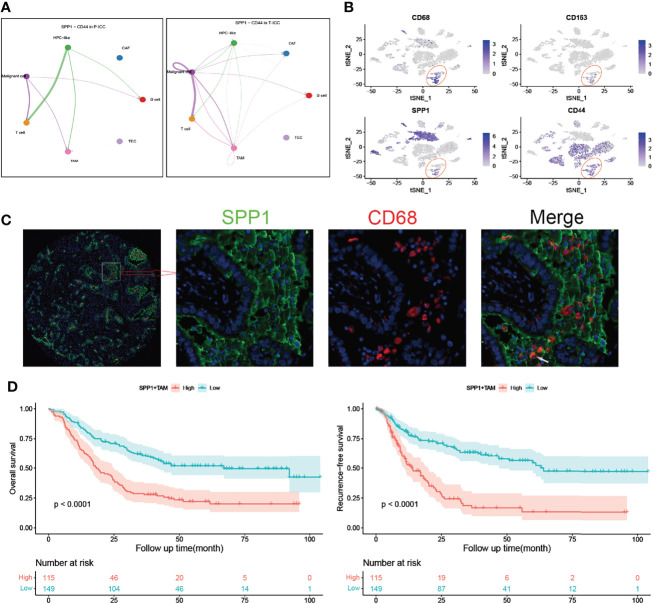
SPP1^+^ TAMs correlate with poor prognosis in ICC. **(A)** Circle plots showing signaling networks of the ligand–receptor (SPP1-CD44) in P-ICC (left) and T-ICC (right). **(B)** t-SNE plots showing the CD68, CD163, SPP1, and CD44 expression levels in all cells of ICC samples. **(C)** Representative microphotographs of SPP1^+^CD68^+^ TAMs. White arrow indicates positive staining of SPP1 and CD68. **(D)** Kaplan–Meier survival curves for OS (left) and RFS (right) of patients with ICC grouped by infiltration levels of SPP1^+^ TAMs. P-values were determined *via* the log-rank test. OS, overall survival; RFS, recurrence-free survival; TAMs, tumor-associated macrophages.

We next performed a DEG analysis of TAMs and found that SPP1 was the highest upregulated gene with a 6.8-fold increase in TAMs following ICBs (**Figure 4A**). APOE and MARCO, which function as pro-M2 polarization and anti-inflammatory genes (40, 41), were also significantly upregulated in T-ICC macrophages. Upregulated genes in T-ICC TAMs were enriched in inflammatory response regulation and complement activation (e.g., TNFA_signaling_via_NFKB, INF_gamma signaling) (**Figure 4B**). Since SPP1 signaling plays a pivotal role in tumor progression (42), we analyzed the SPP1 signaling network in T-ICC and P-ICC tumor ecosystems separately. Intriguingly, new SPP1 signals initiated from TAMS targeting malignant cells or T cells were observed (**Figure 4C**). Different from P-ICC, where SPP1 − (ITGAV+ITGB1) was the dominant L–R pair, SPP1-CD44 was the dominant L–R pair mediating cellular communication in T-ICC (**Figures 4D**, **5A**). Moreover, SPP1^+^ TAMs, C1QC^+^ TAMs, and M2 gene signatures were significantly enriched, while M1 gene signatures were reduced in TAMs after ICB therapy (**Figures 4E, F** **)**.

### SPP1^+^ TAMs Correlate With Poor Prognosis and Immune Infiltrates in ICC

The t-SNE plot demonstrated that SPP1 was highly expressed in malignant cells and TAMs. CD44 showed a high expression in malignant cells, T cells, and TAMs (**Figure 5B**). To quantify the spatial distribution of SPP1^+^ TAMs, we performed dual IF staining in 264 ICC specimens from the ZSH cohort. The typical microphotographs of SPP1^+^CD68^+^ TAMs are presented in **Figure 5C**. The optimal cutoff value for the counts of SPP1^+^ TAMs was 3. Patients with SPP1^+^ TAM counts >3 were considered as high expression and those with counts ≤3 were defined as low expression. Next, the association between SPP1^+^ TAMs and clinical variables was evaluated. As detailed in **Table 1**, high SPP1^+^ TAM infiltration was correlated with elevated serum CA199, CEA, and GGT levels. Patients with high SPP1^+^ TAMs were prone to suffer from lymph node (LN) metastasis, multiple tumors, and larger tumor size (all P < 0.050). Survival analysis revealed that patients with high SPP1^+^ TAMs had significantly shorter OS and a higher recurrence risk (both P < 0.001, **Figure 5D**). Notably, multivariate Cox regression analysis demonstrated that high SPP1^+^ TAM infiltration was an independent prognostic factor for predicting both RFS [HR 2.613 (1.805–3.783), P < 0.001] and OS [HR 1.701 (1.225–2.361), P = 0.002, **Tables 2**, **3**].

**Table 2:** Univariate Cox regression analysis of variables associated with recurrence and overall survival in the ZSH cohort.

Variables	Recurrence		Overall survival	
	HR (95% CI)	*P*	HR (95% CI)	*P*
Sex (male versus female)	1.267 (0.885–1.815)	0.196	1.013 (0.736–1.394)	0.939
Age (> 60 years versus ≤ 60 years)	0.769 (0.545–1.083)	0.133	0.968 (0.709–1.321)	0.837
Liver cirrhosis (yes versus no)	1.209 (0.839–1.741)	0.308	0.929 (0.655–1.319)	0.682
Microvascular invasion (yes versus no)	1.996 (1.381–2.886)	**<0.001**	2.106 (1.520–2.919)	**<0.001**
LN metastasis (yes versus no)	1.527 (0.973–2.395)	0.066	3.357 (2.374–4.748)	**<0.001**
No. of tumors (multi versus single)	1.630 (1.100–2.414)	**0.015**	2.147 (1.530–3.013)	**<0.001**
Tumor size (> 5 cm versus ≤ 5 cm)	2.449 (1.696–3.535)	**<0.001**	1.997 (1.442–2.766)	**<0.001**
Tumor differentiation (II–III versus I–II)	1.082 (0.757–1.547)	0.665	1.335 (0.956–1.865)	0.090
CA199 (>37 U/ml versus ≤37 U/ml)	1.616 (1.136–2.299)	**0.008**	1.951 (1.407–2.706)	**<0.001**
CEA (>5 ng/ml versus ≤5 ng/ml)	1.637 (1.102–2.432)	**0.015**	2.641 (1.903–3.666)	**<0.001**
GGT (>60 U/l versus ≤60 U/l)	1.937 (1.370–2.739)	**<0.001**	2.509 (1.823–3.451)	**<0.001**
AJCC 8th (IIIa–IIIb versus I–II)	1.497 (0.974–2.299)	0.066	3.079 (2.193–4.322)	**<0.001**
SPP1^+^ TAMs (high versus low)	3.050 (2.1344–.361)	**<0.001**	2.359 (1.721–3.234)	**<0.001**

HR, hazard ratio; CI, confidence interval; NA, not available.

Bold indicated statistical significance.

**Table 3:** Multivariate cox regression analysis of variables associated with recurrence and overall survival in the ZSH cohort.

Variables	Recurrence		Overall survival	
	HR (95% CI)	*P*	HR (95% CI)	*P*
Microvascular invasion (yes versus no)	1.571 (1.008–2.449)	**0.046**	1.808 (1.262–2.590)	**0.001**
LN metastasis (yes versus no)	N.A.	N.A.	2.964 (0.909–9.664)	0.072
No. of tumors (multi versus single)	0.926 (0.594–1.444)	0.735	0.990 (0.661–1.482)	0.960
Tumor size (>5 cm versus ≤5cm)	1.719 (1.132–2.611)	**0.011**	1.116 (0.768–1.622)	0.564
CA199 (>37 U/ml versus ≤37 U/ml)	1.271 (0.868–1.861)	0.217	1.474 (1.046–2.077)	**0.027**
CEA (>5 ng/ml versus ≤5 ng/ml)	1.132 (0.734–1.745)	0.574	1.909 (1.346–2.709)	**<0.001**
GGT (>60 U/l versus ≤60 U/l)	1.454 (1.000–2.115)	0.050	1.675 (1.174–2.389)	**0.004**
AJCC 8th (IIIa–IIIb versus I–II)	N.A.	N.A.	0.814 (0.253–2.620)	0.730
SPP1^+^ TAMs (high versus low)	2.613 (1.805–3.783)	**<0.001**	1.701 (1.225–2.361)	**0.002**

HR, hazard ratio; CI, confidence interval; NA, not available.

Bold indicated statistical significance.

**Figure 6:**
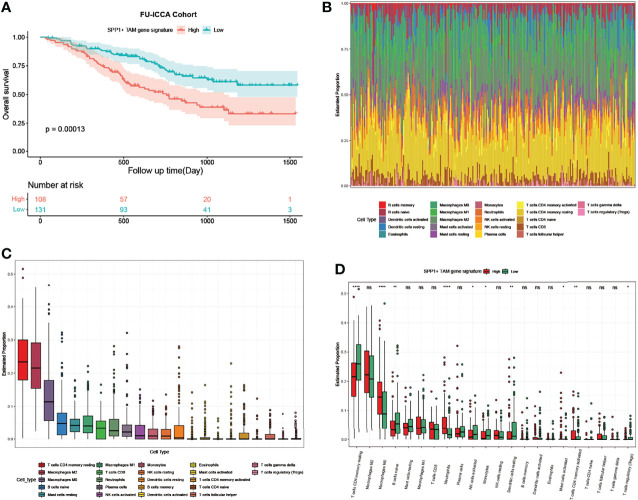
SPP1^+^ TAM gene signature predicts poor prognosis and associates with immune infiltrates in ICC. **(A)** Kaplan–Meier survival curves for OS of the FU-iCCA cohort grouped by the SPP1^+^ TAM gene signature score. **(B)** Stacked bar plots showing 22 immune cell type proportions in 255 ICC samples estimated by CIBERSORT. **(C)** Boxplots showing the ranked infiltration level of each immune cell type in ICC. **(D)** Boxplots showing the ranked infiltration level of 22 immune cell types grouped by the SPP1^+^ TAM gene signature score in ICC. OS, overall survival. *P < 0.05, **P < 0.01, ****P < 0.0001; ns, no significance.

For the bulk RNA-seq data of 255 ICC samples from the FU-iCCA cohort, the mean expression of the top 10 upregulated genes in TAMs following ICBs was defined as the SPP1^+^ TAM gene signature score in the FU-iCCA cohort. The signature score was used to divide them into high- and low-expression groups. Consistently, patients with a high SPP1^+^ TAM gene signature score were associated with poor prognosis (**Figure 6A**). Furthermore, we found a high macrophage infiltration level (M2, M0, M1) in the tumor immune microenvironment using cell-type scores calculated by CIBERSORT (**Figures 6B, C** **)**. This indicates that TAMs constitute a major component of immune cells in ICC and therefore could affect the treatment efficacy of immunotherapy. Also, patients with higher SPP1^+^ TAM gene signatures had significantly lower CD4 memory resting T-cell, higher M0 macrophage, and higher neutrophil infiltration levels compared with those with a lower SPP1^+^ TAM gene signature (**Figure 6D**). Collectively, these data suggest that SPP1^+^ TAMs are associated with unfavorable clinical outcomes in patients with ICC and correlate closely with immune infiltrates in the ICC tumor ecosystem.

## Discussion

Despite promising clinical progress in several cancers, the response rates of ICC to ICBs remain unsatisfactory (10) and the mechanisms underlying ICB resistance are poorly understood. In this study, we assessed the intra-tumoral changes in ICC patients receiving ICBs *via* a publicly available single-cell dataset mining. Our analysis reveals a distinctive cellular ecosystem in ICC following ICB therapy. We observed apparently increased interaction numbers as well as interaction strength between malignant cells and CAFs, suggesting an enhanced crosstalk between these two cell types in response to ICBs. Moreover, VEGF signaling mediated by the PGF–VEGFR1 pair between CAFs and TECs was observed exclusively in the ICB-treated ICC ecosystem. ICC tissues are typically featured with a dominant desmoplastic stroma (43). CAFs have been reported to promote tumor angiogenesis and affect the tumor microenvironment *via* multiple pathways such as IL6/STAT3 (44–46). Our analyses supported that targeting CAFs and the VEGF pathway combined with ICBs might be a rational treatment approach for ICC.

Ligand–receptor pair analysis revealed increased MIF-CD74 signaling, which has been established as a pro-tumorigenic factor (31–34), between malignant cells and TAMs following ICBs. Moreover, blocking this signaling was able to restore the antitumor immune activity to melanoma (35, 36)and thus could be a potential strategy to overcome tumor resistance to ICBs in ICC. Using trajectory inference, we observed that CD4^+^ T cells, especially CD4 KLRB1 (CD161) cells from the ICB-treated group, were predominantly distributed at the terminal ends of the transition trajectory pathway. CD161^+^CD4^+^ T cells could play an immunoregulatory role through cytokine production and were increased in cancer patients compared with healthy individuals (47). Previous studies reported the enrichment of CD161^+^CD4^+^ T cells in the liver during chronic hepatitis (48), and IFN-γ could facilitate liver fibrogenesis by CD161^+^CD4^+^ T cells through the IL-23/IL-17 axis in chronic hepatitis B virus infection (49). In our study, CD161^+^CD4^+^ T cells were denoted as tissue-resident, memory-like T cells due to the high expression of KLRB1, CD40LG, CD69, and ANXA1. This suggests that this CD4^+^ T subtype is closely related with immune response and its role in immunotherapy should be further explored.

TAMs are the main components of the tumor ecosystem and play key roles in the progression of cancers (50, 51). TAMs are classified into pro-inflammatory (M1) or anti-inflammatory (M2) TAMs (52). Differential analysis of TAMs showed that SPP1, well known for its oncogenic role in liver cancer (42, 53, 54), was remarkably upregulated following ICB therapy. Single-cell analyses of colon cancer revealed that TAMs could be divided into C1QC^+^ TAMs and SPP1^+^TAMs. Cell migration, ECM–receptor interaction, and tumor angiogenesis pathways were enriched in SPP1^+^ TAMs, while the complement pathway activation and antigen processing and presentation pathways were enriched in C1QC^+^ TAMs (24). Recently, in a multi-omics analysis, these two TAM gene signatures could stratify cervical patients with different prognoses and patients with C1QC^low^ and SPP1^high^ TAM gene signatures had the worst prognosis in cervical cancer (55). In a single-cell and spatial atlas of colorectal cancer liver metastasis, SPP1^+^ macrophages were specifically present in liver metastatic tumors and responsive neoadjuvant chemotherapy (NAC) could downregulate this subset of macrophages. On the contrary, an increased infiltration level of SPP1^+^ macrophages was observed in non-responsive tumors (56). In our study, SPP1^+^ TAMs, C1QC^+^ TAMs, and M2 gene signatures were enriched in TAMs receiving ICB therapy, whereas M1 gene signatures were enrichment in treatment-naïve TAMs. Furthermore, we explored the clinical value of SPP1^+^CD68^+^ TAMs *via* IF staining in a cohort of 264 patients and it suggested that SPP1^+^ TAMs correlated with adverse clinical outcomes in ICC.

Our current study is mainly based on the analyses of a public single-cell dataset, which inevitably has some limitations and needs further verification. For example, tumor biopsies limited the number of cells for scRNA-seq and the samples were not paired before and after ICB therapy. Also, the five patients receiving ICB therapy could not be further defined as responders or non-responders since the information of ICB efficacy was unavailable.

In conclusion, although further investigations are warranted to validate the findings, our study at least in part unveils the altered landscape in the ICC tumor ecosystem following ICB therapy and highlights the significance of targeting CAFs and SPP1^+^TAMs to guide a more rational immune-based therapy for ICC.

## Data Availability Statement

The datasets presented in this study can be found in online repositories. The names of the repository/repositories and accession number(s) can be found in the article/**Supplementary Material**.

## Ethics Statement

The studies involving human participants were reviewed and approved by the Research Ethics Committee of Zhongshan Hospital. The patients/participants provided their written informed consent to participate in this study.

## Author Contributions

S-JQ, YY, JZ, and JF contributed to the conception and supervision of the study. B-YS and CZ analyzed the data. B-YS, Z-FY, and Z-TW performed the experiments. B-YS, CZ, R-YG, and GL wrote the article. B-YS, R-YG, CZ, and WG prepared the figures and tables. S-JQ and YY critically revised the article. All authors contributed to the article and approved the submitted version.

## Funding

This project is funded by the National Natural Science Foundation of China (No. 82072677, No. 82072672, No. 82103417) and Clinical Research Project of Zhongshan Hospital (Nos. 2020ZSLC62 and 2020ZHZS17).

## Conflict of Interest

The authors declare that the research was conducted in the absence of any commercial or financial relationships that could be construed as a potential conflict of interest.

## Publisher’s Note

All claims expressed in this article are solely those of the authors and do not necessarily represent those of their affiliated organizations, or those of the publisher, the editors and the reviewers. Any product that may be evaluated in this article, or claim that may be made by its manufacturer, is not guaranteed or endorsed by the publisher.

## Abbreviations

ICC, intrahepatic cholangiocarcinoma; ICBs, immune checkpoint blockades; PD, program cell death; HCC, hepatocellular carcinoma; CNV, copy number variation; DEGs, differentially expressed genes; P-ICC, primary ICC; T-ICC, ICBs-treated ICC; BTC, bile tract cancer; scRNA-seq, single-cell RNA sequencing; tSNE, t-distributed stochastic neighbor embedding; GSEA, gene set enrichment analysis; OS, overall survival; RFS, recurrence-free survival; IF, immunofluorescence; CAFs, cancer-associated fibroblasts; TAMs, tumor-associated macrophages; TECs, tumor-associated endothelial cells; HR, hazard ratio; CI, confidence interval.
