# Supplementary material for: Dissecting Intra-Tumoral Changes Following Immune Checkpoint Blockades in Intrahepatic Cholangiocarcinoma via Single-Cell Analysis
Source: Front Immunol. 2022 Apr 26;13:871769. doi: 10.3389/fimmu.2022.871769 (PMC9088915; doi:10.3389/fimmu.2022.871769)
Supplement: Supplementary file 1 [file DataSheet_1.docx]

Supplementary Material

# Supplementary Figures and Tables

**Table S1** Gene signatures used to define subtypes of tumor-associated macrophages

| **Gene Signatures** | **Genes** |
| --- | --- |
| SPP1^+^ TAMs | SPP1, PCSK5, SLC11A1, VCAN, SLC25A37, FLNA, UPP1, BCL6, AQP9, TIMP1, VEGFA, ADM, MARCO, FN1, IL1RN |
| C1QC^+^ TAMs | C1QA, C1QB, ITM2B, C1QC, HLA-DMB, MS4A6A, CTSC, TBXAS1, TMEM176B, SYNGR2, ARHGDIB, TMEM176A, UCP2, CAPZB, MAF, TREM2, MSR1 |
| M1 macrophages | CCL5, CCR7, CD40, CD86, CXCL9, CXCL10, CXCL11, IDO1, IL1A, IL1B, IL6, IRF1, IRF5, KYNU |
| M2 macrophages | CCL4, CCL13, CCL18, CCL20, CCL22, CD276, CLEC7A, CTSA, CTSB, CTSC, CTSD, FN1, IL4R, IRF4, LYVE1, MMP9, MMP14, MMP19, MSR1, TGFB1, TGFB2, TGFB3, TNFSF8, TNFSF12, VEGFA, VEGFB, VEGFC |

TAMs, tumor-associated macrophages

**Table S2** Differentially expressed genes in TAMs before and after ICBs treatment

| **Genes** | **Average log2FC** | **Adjusted *P* value** |
| --- | --- | --- |
| SPP1 | 2.75969445 | 1.82E-06 |
| S100A9 | 2.212815135 | 5.72E-14 |
| NUPR1 | 2.208792344 | 2.57E-05 |
| S100A8 | 2.069038161 | 2.06E-08 |
| RETN | 2.037342857 | 2.17E-07 |
| MARCO | 1.940594128 | 8.53E-12 |
| FCGR3A | 1.777173534 | 5.89E-19 |
| MT2A | 1.733069857 | 3.13E-05 |
| TMEM176B | 1.687229329 | 1.44E-22 |
| APOE | 1.686897175 | 0.001741933 |
| OLR1 | 1.62837721 | 6.68E-24 |
| PLIN2 | 1.594423888 | 0.000210277 |
| APOC1 | 1.538997615 | 9.35E-06 |
| MT1X | 1.538789549 | 5.45E-05 |
| ALOX5AP | 1.495270938 | 9.67E-15 |
| FTL | 1.493783196 | 3.41E-17 |
| CD14 | 1.421949086 | 2.25E-14 |
| GCHFR | 1.371669358 | 3.31E-06 |
| C1QA | 1.315876478 | 3.32E-10 |
| CXCL2 | 1.242855499 | 1.40E-05 |
| S100A4 | 1.234502966 | 9.11E-10 |
| CTSB | 1.228255288 | 3.63E-12 |
| TMEM176A | 1.219764695 | 3.34E-14 |
| MRC1 | 1.199054365 | 0.000713685 |
| CFD | 1.190101682 | 1.89E-11 |
| CXCL3 | 1.166531409 | 0.000216297 |
| C1QB | 1.13217852 | 2.57E-07 |
| VSIG4 | 1.102567779 | 2.17E-11 |
| CTSL | 1.096684822 | 2.49E-11 |
| PLAUR | 1.088582692 | 1.65E-06 |
| VCAN | 1.084195695 | 0.00130891 |
| MNDA | 1.069257489 | 1.33E-06 |
| C1QC | 1.067073002 | 2.85E-08 |
| CD68 | 1.066781764 | 3.24E-14 |
| CTSD | 1.034320799 | 4.20E-15 |
| MS4A4A | 1.011276808 | 1.85E-10 |
| CSTB | 1.011135663 | 6.68E-06 |
| ZEB2 | 1.009547106 | 8.47E-06 |
| CLEC12A | 1.00744877 | 2.02E-06 |
| STMN1 | 0.986618613 | 0.020040224 |
| TXNIP | 0.968046243 | 0.004325029 |
| FN1 | 0.964353511 | 2.22E-06 |
| TSPO | 0.963905392 | 2.13E-12 |
| LINC01272 | 0.940905172 | 6.63E-09 |
| GLUL | 0.933636227 | 1.95E-11 |
| PLTP | 0.932272638 | 0.000474985 |
| SLC11A1 | 0.928465621 | 5.75E-09 |
| RANBP2 | 0.916087143 | 0.000430098 |
| CD63 | 0.914214952 | 2.27E-12 |
| BRI3 | 0.895562521 | 3.33E-05 |
| C5AR1 | 0.891590233 | 2.43E-08 |
| FBP1 | 0.887502416 | 0.03230557 |
| S100A11 | 0.878500582 | 1.94E-13 |
| MT-ND4L | 0.870904361 | 5.79E-09 |
| GPX1 | 0.868078579 | 3.34E-12 |
| FCGR1A | 0.857912086 | 3.75E-06 |
| S100A6 | 0.849518582 | 0.00252645 |
| S100A10 | 0.836637334 | 0.001257358 |
| GRN | 0.821629889 | 1.14E-09 |
| TMEM59 | 0.808106348 | 2.30E-08 |
| GPR65 | 0.806490314 | 8.02E-08 |
| TREM2 | 0.796052918 | 0.000615494 |
| RNF13 | 0.787881741 | 5.74E-07 |
| GPX3 | 0.785547555 | 0.000626255 |
| CD59 | 0.785260759 | 1.00E-04 |
| MS4A7 | 0.784976312 | 8.52E-07 |
| SLC16A3 | 0.784053903 | 2.99E-06 |
| LY96 | 0.747888241 | 1.13E-07 |
| ATP1B3 | 0.746546721 | 0.003194857 |
| SDCBP | 0.73582373 | 1.38E-09 |
| CYBB | 0.734445776 | 3.44E-05 |
| LRPAP1 | 0.732362881 | 0.00392576 |
| GPNMB | 0.730902416 | 1.86E-08 |
| PPT1 | 0.724248682 | 1.29E-05 |
| TREM1 | 0.722102923 | 0.001181762 |
| GSTO1 | 0.72150712 | 0.004402459 |
| ZFYVE16 | 0.714129998 | 0.000136558 |
| FCER1G | 0.711212546 | 2.61E-07 |
| ARHGAP18 | 0.704119834 | 3.09E-06 |
| ATP6AP2 | 0.699133262 | 2.80E-05 |
| SMCO4 | 0.688385324 | 2.50E-05 |
| ARPC5 | 0.683077125 | 9.43E-07 |
| ASAH1 | 0.681272461 | 0.0001162 |
| DMXL2 | 0.680153964 | 0.000315282 |
| RHEB | 0.675386103 | 0.000448026 |
| AIF1 | 0.67292809 | 1.37E-07 |
| TAX1BP1 | 0.670552367 | 0.014856344 |
| RPS4Y1 | 0.655381579 | 6.60E-10 |
| NUP214 | 0.652930367 | 0.000118657 |
| PSAP | 0.650349969 | 1.77E-08 |
| APLP2 | 0.645813865 | 1.56E-07 |
| FCGRT | 0.643557791 | 0.000164241 |
| PTPRC | 0.642646133 | 0.020520388 |
| H2AFJ | 0.638109214 | 0.011839552 |
| FRMD4B | 0.637184246 | 5.46E-06 |
| VAMP8 | 0.631781453 | 3.32E-10 |
| TYROBP | 0.630209618 | 3.02E-10 |
| ANXA5 | 0.626282584 | 0.0009129 |
| SUMO3 | 0.624215978 | 0.009169413 |
| IGSF6 | 0.623707564 | 0.022407778 |
| FNDC3B | 0.615392283 | 0.001300247 |
| RASSF4 | 0.615189744 | 0.001492061 |
| SERPINA1 | 0.613687183 | 7.61E-05 |
| CAST | 0.609260377 | 0.044984536 |
| CAPG | 0.6076362 | 0.000521033 |
| HNMT | 0.607557143 | 0.000455529 |
| KCNMA1 | 0.603541945 | 0.014513398 |
| LAIR1 | 0.603169229 | 0.00057532 |
| TMED9 | 0.598534171 | 2.72E-05 |
| SNX2 | 0.597750271 | 4.83E-05 |
| DNASE2 | 0.597175855 | 0.007200699 |
| DDX24 | 0.595362875 | 0.002688585 |
| DNMT1 | 0.593970705 | 0.004421765 |
| CD99 | 0.592419252 | 0.008249653 |
| C3 | 0.584328156 | 1.35E-06 |
| IFI16 | 0.57987066 | 0.003272947 |
| CTSA | 0.579441941 | 0.000685583 |
| ANXA2 | 0.577201291 | 4.44E-05 |
| SLC7A7 | 0.57678799 | 2.45E-05 |
| PMP22 | 0.575582102 | 0.004561194 |
| C3AR1 | 0.573273247 | 0.024956425 |
| ELL2 | 0.568874043 | 0.001807483 |
| DAB2 | 0.567677617 | 0.015715391 |
| CTSC | 0.566427826 | 2.96E-06 |
| DDX3Y | 0.565242399 | 0.000218927 |
| MAFB | 0.564441837 | 0.000136403 |
| ALCAM | 0.563669491 | 0.004610279 |
| RTN3 | 0.563457473 | 7.28E-05 |
| LGALS1 | 0.562016887 | 0.001930423 |
| PRNP | 0.561313078 | 0.014268344 |
| LILRB4 | 0.547404974 | 0.000274537 |
| TNFSF10 | 0.539021555 | 0.00068151 |
| ITM2B | 0.537591059 | 0.003268397 |
| SAMHD1 | 0.536753696 | 0.007435468 |
| LINC01094 | 0.535849225 | 6.14E-07 |
| MORF4L1 | 0.534548967 | 1.19E-05 |
| HP1BP3 | 0.53390566 | 0.021740833 |
| ASGR1 | 0.5305365 | 0.001751663 |
| PTTG1IP | 0.527211045 | 0.001132132 |
| ST3GAL6 | 0.52418948 | 0.004656701 |
| LINC00998 | 0.51983584 | 0.004656536 |
| VAPA | 0.516836908 | 0.003110208 |
| TGOLN2 | 0.515166775 | 0.01180271 |
| AGTRAP | 0.512578345 | 0.002210575 |
| TMEM50A | 0.510081452 | 0.003233335 |
| ITGAM | 0.50695429 | 0.005484786 |
| ATP6V0E1 | 0.505237417 | 0.000954448 |
| LAPTM5 | 0.504406669 | 0.000585882 |
| CLEC2B | 0.502503646 | 0.012634533 |
| UBE2D1 | 0.501872335 | 0.01639974 |
| ATP6V1F | 0.500619745 | 0.002835154 |
| ITPR2 | 0.499016173 | 0.004078595 |
| PGD | 0.498019682 | 0.001347579 |
| SH3BGRL | 0.496565861 | 0.034187534 |
| GPR34 | 0.493300901 | 0.000368164 |
| SERF2 | 0.482946278 | 2.09E-05 |
| RTN4 | 0.480977955 | 0.021748873 |
| PYCARD | 0.476593138 | 0.00446906 |
| PLXDC2 | 0.474757325 | 0.009278136 |
| TMEM230 | 0.472383478 | 0.024683537 |
| NDUFB11 | 0.45971604 | 0.010863916 |
| FAM105A | 0.453938457 | 0.001065456 |
| NDUFB7 | 0.450874214 | 0.018016108 |
| SLCO2B1 | 0.44755438 | 0.012386817 |
| GNPDA1 | 0.446070798 | 0.003737831 |
| LRP1 | 0.43620463 | 0.000673468 |
| RNF7 | 0.431967753 | 0.046419144 |
| NGFRAP1 | 0.431109746 | 0.010539213 |
| AP2S1 | 0.429154441 | 0.013367644 |
| PCMT1 | 0.425363865 | 0.001953597 |
| KCTD12 | 0.423260398 | 0.049647735 |
| HLA-DRB5 | 0.420220157 | 0.005179128 |
| BST2 | 0.418777491 | 0.002633877 |
| NPC2 | 0.415967152 | 0.002735541 |
| CD151 | 0.408870891 | 0.004380773 |
| DYNC1I2 | 0.404325576 | 0.007674269 |
| ATP6V0B | 0.398634663 | 0.000970198 |
| OAZ1 | 0.39329416 | 0.00019736 |
| TMSB4X | 0.386612978 | 2.98E-05 |
| MFSD1 | 0.367431483 | 0.023427963 |
| APBB1IP | 0.328184878 | 0.020401383 |
| CYBA | 0.327674832 | 0.00134482 |
| RPLP1 | -0.321932476 | 0.028155272 |
| RPS2 | -0.351592676 | 0.00263203 |
| RPS19 | -0.359447026 | 0.000148879 |
| RPL27A | -0.385502063 | 0.003167753 |
| RPL24 | -0.387506438 | 0.00445352 |
| RPS25 | -0.403426964 | 0.01174449 |
| HLA-DQB2 | -0.408477906 | 0.000295409 |
| RPS15 | -0.411517277 | 6.64E-06 |
| RPL13 | -0.41439828 | 4.39E-05 |
| RPL11 | -0.421592126 | 2.73E-06 |
| NACA | -0.423378925 | 0.010734998 |
| RPL14 | -0.428219434 | 0.005006396 |
| RPL13A | -0.429102642 | 0.000946713 |
| RPL10 | -0.429347746 | 1.11E-07 |
| RPL28 | -0.434591321 | 6.39E-07 |
| RPL38 | -0.440341894 | 1.92E-06 |
| RPL39 | -0.444130698 | 0.000290917 |
| RPS16 | -0.44882312 | 4.10E-05 |
| RPL8 | -0.455565697 | 9.72E-06 |
| RPS27A | -0.484260064 | 1.78E-06 |
| RPS3 | -0.489501889 | 1.18E-05 |
| RPS20 | -0.49643872 | 3.95E-05 |
| RPL35 | -0.498522261 | 2.27E-10 |
| RPS23 | -0.502821136 | 7.46E-05 |
| RPS8 | -0.510510907 | 0.000180611 |
| RPL18 | -0.511865996 | 9.70E-06 |
| GNB2L1 | -0.513717536 | 1.66E-05 |
| RPS28 | -0.51699903 | 3.47E-13 |
| RPL7A | -0.520677047 | 1.45E-06 |
| RPLP2 | -0.523629199 | 9.29E-09 |
| RPSA | -0.535170935 | 0.000659811 |
| RPL5 | -0.536453152 | 0.000231479 |
| RPS12 | -0.538477723 | 4.20E-05 |
| RPL7 | -0.539170392 | 0.000308134 |
| EEF1B2 | -0.548241143 | 0.046195938 |
| RPL29 | -0.551078272 | 2.22E-06 |
| RPS14 | -0.557001687 | 2.57E-10 |
| RPL6 | -0.561730523 | 3.28E-06 |
| RPS6 | -0.565397811 | 2.03E-07 |
| RPL30 | -0.569409158 | 6.94E-06 |
| RPL19 | -0.569485655 | 3.09E-08 |
| ACTG1 | -0.580965001 | 0.002664822 |
| RPL22 | -0.585000651 | 1.16E-06 |
| PABPC1 | -0.587339984 | 4.35E-05 |
| RPL21 | -0.590662828 | 1.05E-10 |
| RPS5 | -0.593982148 | 5.19E-08 |
| RPL10A | -0.600180956 | 3.03E-09 |
| RPL26 | -0.604721277 | 5.02E-12 |
| RPL35A | -0.616432965 | 2.65E-10 |
| RPS7 | -0.624263129 | 5.13E-10 |
| RPL3 | -0.626496883 | 2.04E-09 |
| RPLP0 | -0.626532375 | 0.000215763 |
| RPL12 | -0.632405796 | 1.52E-12 |
| RPL32 | -0.633209298 | 4.68E-13 |
| RPL37A | -0.641010823 | 9.22E-14 |
| RPL31 | -0.642907685 | 1.23E-08 |
| RPS18 | -0.647785561 | 2.67E-11 |
| EEF2 | -0.657511443 | 0.027471813 |
| RPL18A | -0.657648827 | 6.01E-10 |
| RPS21 | -0.658188504 | 6.46E-10 |
| CNN2 | -0.673592431 | 1.15E-05 |
| RPS11 | -0.690088303 | 4.15E-11 |
| RPS3A | -0.69305055 | 8.28E-15 |
| MALAT1 | -0.69859855 | 2.78E-07 |
| RPL34 | -0.703936225 | 2.01E-12 |
| RPS27 | -0.704485362 | 1.43E-13 |
| RPS13 | -0.724540037 | 7.73E-12 |
| RPL9 | -0.735247975 | 4.34E-14 |
| RPL23A | -0.738370939 | 3.03E-09 |
| RPL4 | -0.738953654 | 6.81E-09 |
| RPL41 | -0.74382718 | 3.03E-16 |
| ZNF90 | -0.75139494 | 3.94E-05 |
| RPS15A | -0.755851703 | 1.85E-16 |
| EEF1A1 | -0.760043747 | 8.59E-14 |
| RPS17 | -0.763711922 | 8.20E-16 |
| RPL36 | -0.799931807 | 5.97E-22 |
| EIF5A | -0.81298282 | 0.000365158 |
| RPS4X | -0.865919138 | 1.96E-14 |
| CFP | -0.878882499 | 0.003279016 |
| RPL37 | -0.89132887 | 4.05E-22 |
| NBEAL1 | -0.937113331 | 0.000184168 |
| IGHA1 | -0.947553312 | 4.61E-06 |
| RPS10 | -0.950687266 | 7.14E-15 |
| RPL36A | -1.027490795 | 7.61E-17 |
| CLEC10A | -1.061071427 | 0.003433928 |
| RPS29 | -1.071584225 | 1.63E-25 |
| MARCKSL1 | -1.083035601 | 0.000411843 |
| RPL17 | -1.100688546 | 2.31E-13 |
| AC090498.1 | -1.120508545 | 4.00E-09 |
| HSPA1B | -1.220201903 | 0.000280432 |
| XIST | -1.2268446 | 6.88E-17 |
| HLA-DQA2 | -1.318582027 | 1.29E-07 |
| BIRC3 | -1.553228092 | 2.34E-06 |
| HBA1 | -1.663939449 | 1.69E-05 |
| HBA2 | -1.664125043 | 3.24E-12 |
| LGALS2 | -2.039119528 | 2.32E-22 |
| HBB | -2.518770111 | 3.43E-07 |

## Supplementary Figures


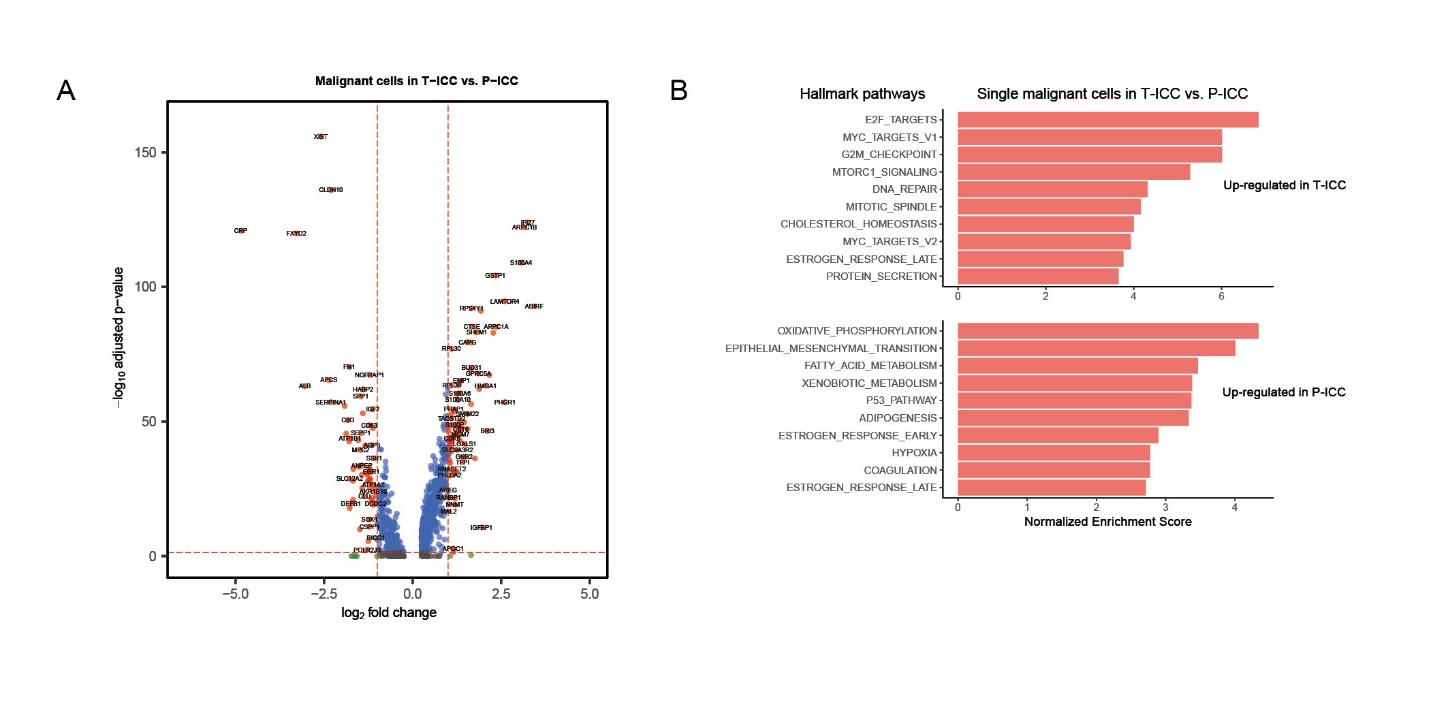


**Supplementary Figure 1.** Differential analysis of single malignant cells in P-ICC and T-ICC

(A) Volcano plot showing differentially expressed genes of malignant cells between P-ICC and T-ICC. (B) Bar chart showing the upregulated Hallmark gene set in T-ICC and P-ICC, respectively. P-ICC, primary ICC; T-ICC, ICBs-treated ICC
